# A Hardware Implemented Autocorrelation Technique for Estimating Power Spectral Density for Processing Signals from a Doppler Wind Lidar System

**DOI:** 10.3390/s18124170

**Published:** 2018-11-28

**Authors:** Sameh Abdelazim, David Santoro, Mark Arend, Fred Moshary, Sam Ahmed

**Affiliations:** 1 The School of Computer Sciences and Engineering, Fairleigh Dickinson University, Teaneck, NJ 07666, USA; 2 Electrical Engineering Department, The City College of the City University of New York, New York, NY 10031, USA; dsantoro@ccny.cuny.edu (D.S.); marend@ccny.cuny.edu (M.A.); fmoshary1@gmail.com (F.M.); ahmed@ccny.cuny.edu (S.A.)

**Keywords:** Doppler Lidar, remote sensing, wind Lidar, FPGA, autocorrelation

## Abstract

A signal processing technique utilizing autocorrelation of backscattered signals was designed and implemented in a 1.5 µm all-fiber wind sensing Coherent Doppler Lidar (CDL) system to preprocess atmospheric signals. The signal processing algorithm’s design and implementation are presented. The system employs a 20 kHz pulse repetition frequency (PRF) transmitter and samples the return signals at 400 MHz. The logic design of the autocorrelation algorithm was developed and programmed into a field programmable gate array (FPGA) located on a data acquisition board. The design generates and accumulates real time correlograms representing average autocorrelations of the Doppler shifted echo from a series of adjustable range gates. Accumulated correlograms are streamed to a host computer for subsequent processing to yield a line of sight wind velocity. Wind velocity estimates can be obtained under nominal aerosol loading and nominal atmospheric turbulence conditions for ranges up to 3 km.

## 1. Introduction

The utilized LIDAR technique is based on the detection and analysis of backscattered light resulting from a laser beam’s interaction with aerosol particles and constituents. The technique is used in many atmospheric measurements and applications [1,2]. Coherent Doppler Lidar (CDL) systems are widely used to make atmospheric measurements as the measurements can be remote, real time, and of high spatial resolution under clear air conditions [3]. They have been widely adopted in applications such as measuring atmospheric wind velocity, turbulence, aerosol concentration, cloud height, cloud velocity, and detection of atmospheric constituents as well as pollutants. An early CDL system for wind sensing was reported in 1970 by Huffaker et al. [4] and employed a 10.6 µm CO_2_ CW laser. Operating a LIDAR system at shorter wavelengths allows for higher spectral and velocity resolution. Systems operating at wavelengths shorter than 2 µm have been widely used [5,6,7,8].

The CDL system described in this paper utilizes two acousto-optic modulators (AOMs) in series where each shifts Local Oscillator (LO) signal by 42 MHz resulting in a total shift of 84 MHz. The pulse repetition frequency (PRF) is 20KHz. Received backscattered signals are sampled by a 400 MHz, 14-bit analog-to-digital converter (ADC). Table 1 summarizes system’s specifications. The instrument was designed and developed at the Remote Sensing Laboratory of the City College of New York (CCNY), where several remote sensing instruments such as multi-wavelength direct detection Lidar, ceilometer, and sun-photometer are concurrently operated to collect optical parameters of atmospheric aerosols [2]. The present system specifications and components’ detailed descriptions were reported previously in [9]. In addition, the paper discussed the use of velocity estimators for the frequency of the peak spectral density that were estimated by calculating and accumulating periodograms. These periodograms were obtained by calculating the square modulus (power) of the Fast Fourier Transform (FFT) of the time-domain backscattered signals [10]. 

An alternative technique to the estimation of periodograms was presented previously [11]. This alternative technique involves the calculation of the autocorrelation of backscattered time-domain received signals’ correlograms, for a number of lags. Correlograms are accumulated for 10,000 received pulses before they are used to generate a power spectrum, which allows for spatial resolution adjustment unlike the FFT technique. A spatial resolution adjustment is achieved by calculating the autocorrelation of backscattered signals and then calculating power spectra for any desired range gates. Received signals in a correlogram, which are real, are converted into complex representations. Power spectra, which are real, are estimated by calculating the FFT of correlograms, i.e., they are Fourier transform pair. Since power spectra are real, correlograms must be complex Hermitian (real part even, imaginary part odd). A correlograms’ complex representation is achieved by complex demodulation of received signals [12,13,14,15,16].

## 2. Autocorrelation (Analog Complex Demodulator) Algorithm

In heterodyne optical detection, local oscillator and backscattered signals are optically mixed through an optical coupler. The resulting mixed signal is then incident upon a balanced photodetector. The power spectrum of received signals is found by calculating the FFT of the autocorrelation as shown in Equations (1) and (2):

(1) $$R\left(\tau \right)=\overset{\infty }{\underset{-\infty }{\int }}f\left(t\right)f\left(t+\tau \right)dt$$

(2) $$G\left(f\right)=\overset{\infty }{\underset{-\infty }{\int }}R\left(\tau \right)e^{-j2\pi f\tau }\;dt$$

where; f(t) represents time domain signals, R(τ) is the signals’ autocorrelation, and G(f) is the Fourier transform (power spectrum).

**Figure 1:**
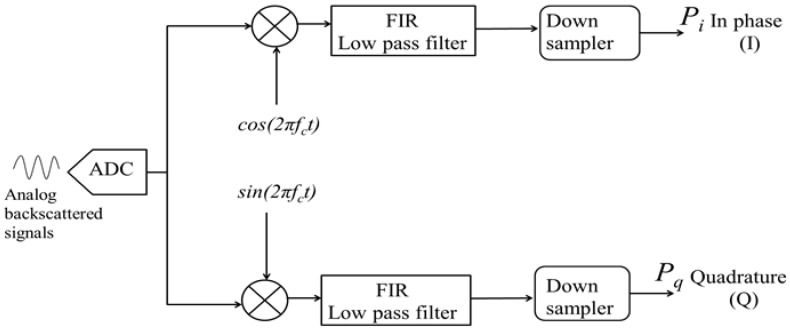
Autocorrelation algorithm block diagram in which input signals are split into two paths; One path is multiplied by a cosine to produce an in-phase (I) signal and the other path is multiplied by a sine to produce a quadrature (Q) signal. A FIR low pass filter is used to filter out the high frequencies of both the I and Q components before being down sampled.

In the autocorrelation technique, digitized received signals are split into two paths (Figure 1). The upper signal path is mixed with a cosine signal oscillating at 84 MHz to produce an in-phase (I) component; The lower signal path is mixed with a sinusoidal signal oscillating at 84 MHz to produce a quadrature (Q) component [17,18]. Mixing the received signals (oscillating at 84 MHz +/− Doppler shift) with an 84 MHz cosine and sine signals produces two output signals, a high frequency (sum of the two frequencies) component and a low frequency (difference of the two frequencies) component. A low-pass finite impulse response (FIR) filter is used on each path to eliminate unwanted high frequency components. Filtered signals’ low frequency components are then down-sampled by a factor of four, which increases the original sampling period from 2.5 ns (400 MHz) to 10 ns (100 MHz).

The resulting complex time sequence *S*(*n*) = *P_i_*(*n*) + *j P_q_*(*n*) is then applied to an M-lag autocorrelation circuit, which computes an autocorrelation matrix *D*(*m,n*) = S^*^(*n*)·*S*(*n* + *m*) for m = 0 to M-1(lags) and n = 0 to N − 1 (number of time domain samples), where S^*^ = *p_i_*(*n*) − *j p_q_*(*n*) is the complex conjugate of *S*(*n*). The calculation process repeats for 10,000 laser shots and the elements of the D matrix are accumulated and streamed to a host computer. Once the accumulated lags’ matrix (Equation (3)) is streamed to the host computer, further processing is conducted to calculate the power spectrum of received signals:

(3) $$D=\left[\begin{array}{cccccc} S_{0}S_{0}^{*} & S_{0}S_{1}^{*} & S_{0}S_{2}^{*} & \ldots & \ldots & S_{0}S_{M-1}^{*}\\ S_{1}S_{1}^{*} & S_{1}S_{2}^{*} & S_{1}S_{3}^{*} & \ldots & \ldots & \vdots \\ S_{2}S_{2}^{*} & S_{2}S_{3}^{*} & S_{2}S_{4}^{*} & \ldots & \ldots & \vdots \\ \vdots & \vdots & \vdots & \ldots & \ldots & \vdots \\ S_{n-1}S_{n-1}^{*} & S_{n-1}S_{n-2}^{*} & 0 & \ldots & \ldots & \vdots \\ S_{n}S_{n}^{*} & 0 & 0 & \ldots & \ldots & \vdots \end{array}\right]$$

where; *M* is the number of lags, *n* is the number of acquired samples, *S* and S^*^ denote to an autocorrelation sample and its complex conjugate, respectively.

To calculate the power spectrum of a certain range gate, the columns of the *D* matrix are accumulated from the *i*th row to the *j*th row, where *i* and *j* are the first and last corresponding samples of that range gate, respectively. This accumulation process produces a 1×M autocorrelation vector, which is complex (in-phase and quadrature components). Since the autocorrelation is symmetric, we construct the second half of the autocorrelation vector by making its real part even and imaginary part odd, Hermitian. Finally, we find the power spectrum of that range gate’s signals by calculating the FFT of the constructed complex autocorrelation vector. 

## 3. Selection of Number of Autocorrelation Lags and Down Sampling Factor

In order to determine the optimal number of autocorrelation lags and the down sample factor that is required to obtain the highest SNR, time-domain atmospheric backscattered signals from 10,000 pulses, were processed using both the FFT and autocorrelation (with different number of lags, and different down sample factors) techniques. 

Power spectra of time-domain atmospheric backscattered signals were calculated using the FFT technique and were compared against those calculated using the autocorrelation technique with the following parameters: (a) number of lags = 16 and down sampling factor = 4, (b) number of lags = 8 and down sampling factor = 4, (c) number of lags = 4 and down sampling factor = 4. Down sampling digitized signals, originally sampled at 400 MHz, by a factor of 4 reduces the sampling rate to 100 MHz, which reduces the maximum detectable Doppler shift to 50 MHz, i.e., maximum obtained wind velocity is 38 m/s. On the other hand, down sampling by a factor of 2 reduces the sampling rate to 200 MHz, which allows for detecting Doppler shifts up-to 100 MHz, i.e., wind velocity up-to 79 m/s. However, a higher spectral resolution results in a lower spatial resolution [19]. 

**Figure 2:**
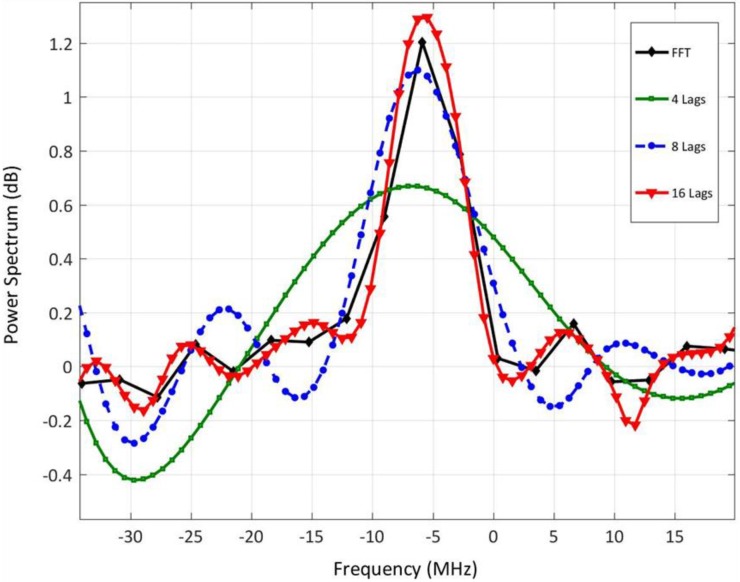
Power spectra of backscattered signals calculated by both FFT and autocorrelation with 16, 8, and 4 lags.

Figure 2 shows the power spectra of backscattered signals estimated by calculating the FFT, and autocorrelation of 16, 8 and 4-lags. It can be shown that the 16-lags’ power spectra has a higher SNR than that of 8 and 4 lags. It is worth noting that even when using 8-lags, frequency resolution could be finer by increasing the time delay between lags, i.e., (τ) of the autocorrelation. Increasing τ, which is achieved by increasing the down sampling factor, results in a finer frequency resolution. However, this results in a reduction in the maximum measured frequency. Table 2 summarizes all analyzed cases of number of lags, down sampling factors, and different values of τ. Choosing 16-lags results in a frequency resolution of 3.2 MHz (i.e., velocity resolution ~2.4 m/s), whereas 8-lags results in a frequency resolution of 6.7 MHz (i.e., velocity resolution of ~4.8 m/s).

The sampling period of the 400 MSPS is 2.5 ns. The frequency resolution of the FFT of the backscattered data is calculated as, $f_{res}=f_{s}/N$, where *f_s_* is the sampling frequency and *N* is the number of samples.

It is clear from Table 2 that the 16-lags case with τ = 10 ns provides the highest velocity resolution with a reasonable maximum velocity range (38 m/s). However, the 8-lags with τ = 10 ns design was selected due to limitations in logic circuit resources on the FPGA. It also provides a better velocity resolution than 8-lags with τ = 15 ns. Technology improvement can allow for increasing number of lags and reducing lag delays, i.e., pre-processing a larger number of samples. 

## 4. Logic Design and FPGA Programming Implementation

In this section, the I-Q generator, low-pass filter, down sampler, autocorrelation, and accumulator digital circuits as designed and implemented on the FPGA are presented. 

### 4.1. Input Signals In-Phase (I) and Quadrature (Q) Generation Digital Circuit

This logic circuit generates the in-phase signal component by multiplying the input time domain digitized signals (8 k sample vector) by an 8 k sample vector consisting of cos(2πf_c_), where the AOM frequency shift (*f_c_*) is 84 MHz. Similarly, the quadrature (Q) component is generated by multiplying the input time domain digitized signals by an 8 k sample vector consisting of sin(2πf_c_).

### 4.2. Low-Pass (FIR) Filter Digital Circuit

The function of this circuit is to filter out the high frequency signals of both in-phase and quadrature components. The FIR cut off frequency is approximately 50 MHz.

### 4.3. Down Sampler Circuit

A down sampler circuit reduces filtered signals size by a factor of 4. For every four received samples, it ignores three samples and sends the fourth one to the next logic circuit. As a result, the filtered time domain in-phase and quadrature signals are reduced in size from 8 k to 2 k samples.

### 4.4. Autocorrelation Digital Circuit

**Figure 3:**
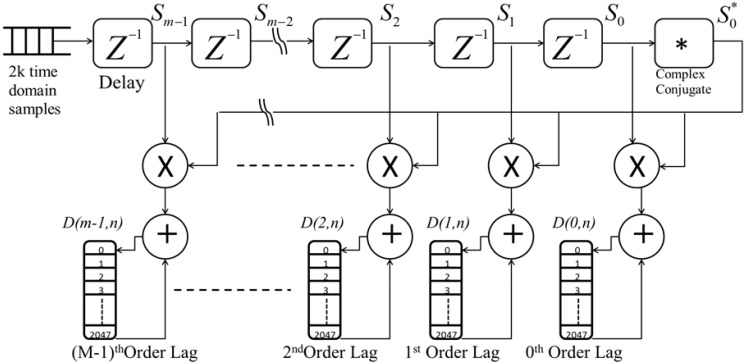
Autocorrelation circuit block diagram as implemented on the FPGA in which input vector is delayed through time-delay circuits and then the complex conjugate of the complex I-Q signals is computed and multiplied by the tapped delayed I-Q samples that produces the autocorrelation lags. These lags are then accumulated through the accumulator circuits for 10,000 laser shots.

The function of the autocorrelation logic circuit is to calculate the autocorrelation of the input signals and accumulate the resulting lags-matrix. Digitized, filtered and down converted input signals arrive at the autocorrelation logic circuit as a 2 k samples vector. This vector passes through M-time delay logic elements, Figure 3, to calculate 0 to M-1 lags. An input vector is tapped at each delay output. A complex conjugate generator logic circuit is placed right after the last delay element to output the complex conjugate d^*^ = *d_i_*(*n*) − *j d_q_*(*n*) of the input signal. The output of the complex conjugate is then multiplied by tapped output of each delay element, resulting in *D*(*m,n*) = d^*^(*n*)·*d*(*n + m*), where, *n* is the sample number, and m is the delay (lag) number. As a result, m vectors are generated where each has 2 k sample length. These 2 k sample vectors are fed to accumulation logic circuits to be accumulated for 10 k laser shots. 

## 5. Vertical Wind Velocity Measurements

In this section, vertical wind velocity is presented. A direct detection aerosol Lidar using an Nd-YAG laser at 1064 nm was operated while operating the CDL system. These Lidar systems were operated on the campus of the City College of New York (CCNY), New York, NY, USA (latitude: 40°49’ N, longitude: 73°56’ W). As the state of the atmospheric boundary layer was changing, it was useful to characterize and baseline this change with the operation of the two Lidar systems. The velocity is estimated by fitting the power spectrum to a Gaussian curve and calculating its moments.

**Figure 4:**
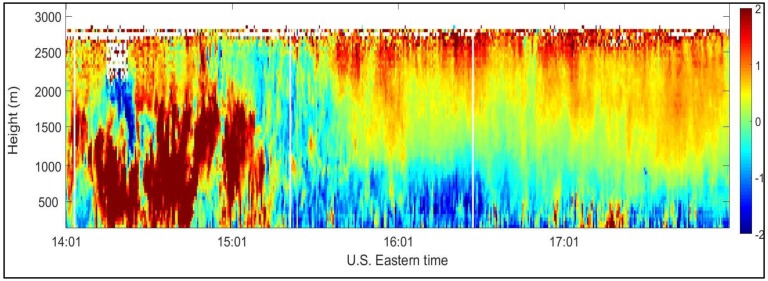
Vertical wind velocity (m/s) vs. time and height measured at the CCNY remote sensing Laboratory between 14:01–18:01 p.m. EDT on 12 July 2012.

Estimating backscattered signals power using an autocorrelation technique allows for varying spatial resolution, which allows for estimating wind velocity when the SNR is very low. For example, a range resolution of 48 m, equivalent to the length of the laser pulse, is chosen at low altitudes where SNR is high. At higher altitudes, when the SNR is very low and wind velocity cannot be reliably estimated, the range resolution can be increased to an arbitrary length to allow for accumulating more of the signals’ power. Figure 4 shows vertical wind velocities (12 July 2012, 14:01 and 18:01 EDT [20,21]) vs. time and height that were estimated using 48 m range resolution. It is clear that wind velocity could be measured up to approximately 2.7 km. 

**Figure 5:**
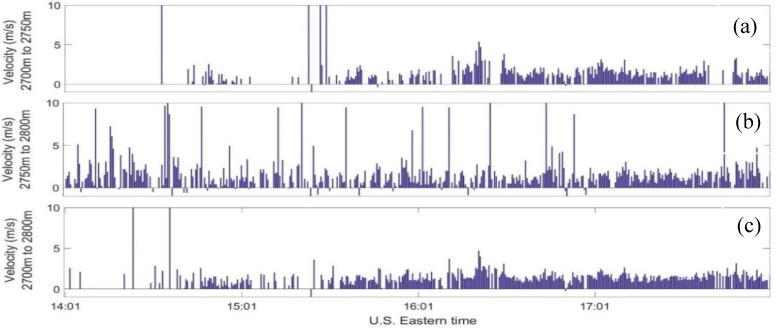
Wind velocity estimated from 2700 m to 2750 m (**a**) and from 2750 m to 2800 m (**b**) using a spatial resoltuion of 48m and from 2700 m to 2800 m (**c**) using a 96m spatial resolution vs. time measured at the CCNY Remote Sensing Laboratory between 14:01–18:01 p.m. EDT on 12 July 2012.

Figure 5a,b shows the estimated vertical wind velocity from two range gates of 48 m each, vs. time extending from 2700 m to 2750 m and from 2750 m to 2800 m, respectively. Figure 5c shows the estimated vertical wind velocity from a single 96 m range gate extending over the height of the earlier two gates combined, i.e., from 2700 m to 2800 m. It is clear from the two 48 m range gates plots in Figure 5a,b, that the estimated wind velocity is not consistent. This is indicated by large velocity magnitudes, spikes, surrounded by relatively smaller magnitudes. Spikes are present when the SNR is low. This occurs when the aerosol concentration is low, usually at high altitudes. The quality control procedure applied on the data is to accept SNR that is above a pre-defined threshold. However, when the threshold is very low, noise can mistakenly be picked up by the velocity estimator and produce invalid wind velocity. Processing the same signals with a spatial resolution of 96 m results in wind velocities that are more consistent and have a lower number of spikes. It is worth noting that the lack of wind velocity estimation from 2700 m to 2750 m between 14:01 to approximately 15:01 (EDT), which was caused by SNR below a threshold value, was populated with more estimates at the next range gate, 2750 m to 2800 m. When processing signals from the 96 m range gate at the same period of time, not many consistent estimates were carried out. In fact, it shows two spikes within a large time period of no estimates. This can be explained as having very low SNR at gate (a) followed by bad signals at range gate (b). When these two range gates were combined and processed, they did not produce valid estimates (Figure 5c). One can propose a new technique in estimating wind velocity by using an adaptive range gating which can automatically vary a range gate’s height based on the measured SNR and also analyzes continuous gates rather than discrete gates. 

## 6. Discussion

**Figure 6:**
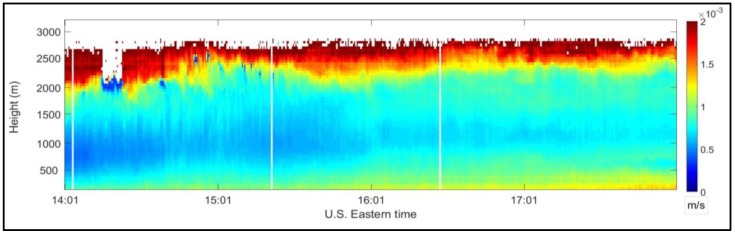
The CRLB on variance of wind velocity estimate vs. time and height measured at the CCNY Remote Sensing Laboratory between 14:01–18:01 p.m. EDT on 12 July 2012.

Increasing range gate size allows for integrating more signal power, which results in improvement in Cramer Rao Lower Bound (CRLB) on the variance of wind velocity estimates. However, accumulating more received power from a longer range gate and/or measurement time may result in scatterers that do not have a constant distribution or velocity across the chosen range gate, which means that the variance of estimates might not improve without limit as more power is accumulated [22]. The CRLB is related to signal power by the following equation [9,22,23]:

(4) σCRLB=2π4(fFs)32δKm(1+0.16Fsδf)

where, δ is the wideband SNR for a single return data sample, K is the number of accumulated power spectra, m is the number of spectral channels (frequency bins), and (*f*/*F_s_*) is the spectra second moment width normalized to the sampling frequency = 0.04. A low CRLB indicates a high precision of wind velocity estimation. Figure 6 shows the CRLB on the variance of wind velocity estimates measured on 12 July 2012. It is clear that wind velocity could be estimated with a precision of approximately 0.001 m·s^−1^ for as high as 2 km. At higher altitudes where the SNR is very low, the CRLB increases to approximately 0.002 m·s^−1^. 

**Figure 7:**
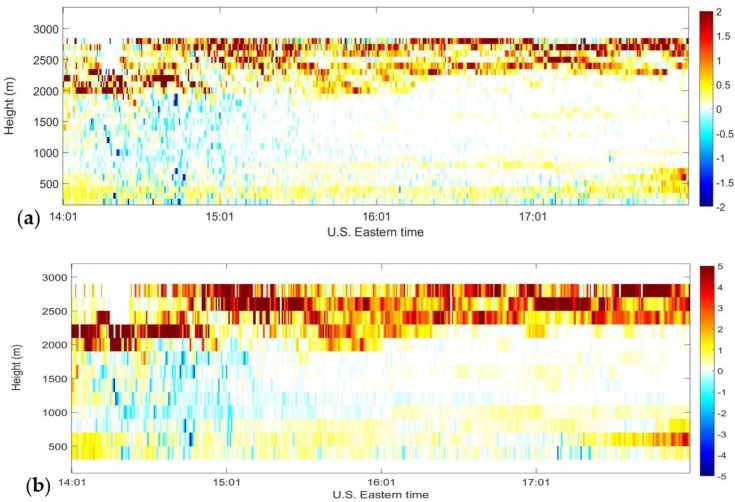
Percentage change in the CRLB (**a**) 92 m vs. 48 m range resolution, and (**b**)192 m vs. 48 m range resolutions vs. time and height measured at the CCNY Remote Sensing Laboratory between 14:01–18:01 p.m. EDT on 12 July 2012.

To examine the effect of varying range resolution on velocity estimation precision, correlograms of backscattered signals measured on 12 July 2012 were processed three times using the following range gate resolutions: 48 m, 96 m, and 192 m. The CRLB on variance of wind velocity estimate was calculated for each case. The percentage of improvement of the CRLB when increasing range resolution from 48 m to 96 m is shown in Figure 7a whereas Figure 7b shows the same percentage when increasing range resolution from 48 m to 192 m. Both Figure 7a,b shows an increase in the velocity estimate precision especially at higher altitudes where the SNR is very low. Increasing range resolution from 48 m to 96 m improves the CRLB by as much as 2% whereas increasing the resolution from 48 m to 192 m improves the CLRB by 5%. These percentage improvements illustrate that the autocorrelation technique is advantageous over the FFT technique where the range size of periodograms is fixed. It is worth noting that in the FFT technique, a range gate size can be extended by integer increments of the original FFT gate size; the overlap of range gates can be varied by changing the step size between successive FFTs—at a price in latency, memory, and processing power. However, the autocorrelation technique has the advantage of allowing for any desirable range gate size.

## 7. Conclusions

An autocorrelation signal processing algorithm of a CDL system for wind measurement was designed and implemented. The algorithm calculates and accumulates received signals’ correlograms by finding the in-phase and quadrature components, down sampling, and calculating the autocorrelation of received signals. A logic design of the autocorrelation algorithm was created and programmed on a FPGA to generate and accumulate real time correlograms representing average autocorrelations of the Doppler shifted echoes from a series of range gates, where range gates can be varied in length. The signals’ power is then found by calculating the fast Fourier transform of the accumulated correlograms. Atmospheric measurements of a direct detection aerosol Lidar were used to validate atmospheric measurements of the CDL by simultaneously operating the two Lidars. The autocorrelation technique has the advantage of varying the range gate in real time. This allows for adaptive signal processing so that performance of the Lidar system can be adjusted depending on the changing atmospheric conditions. Choosing the best temporal and spatial sampling strategies requires that tradeoffs be made in real time. Changing range resolution allows for improving wind velocity estimates by increasing the SNR of backscattered signals. It was found that doubling the range gates at high altitudes where the SNR is very low improved the CRLB on velocity estimate variance by as much as 5%. New signal processing techniques could be implemented where a sliding range gate would be used to calculate signals’ power in the areas where the SNR is low instead of using predefined discrete gates thus improving the accuracy of the wind velocity calculations.

## Figures and Tables

**Table 1:** System specifications.

Parameter	Value
Local Oscillator’s Power	500 mW
Wave Length	1545.2 nm
LO linewidth	~3 KHz
Laser Type	Fiber-optics
Detector’s Bandwidth	125 MHz
Optical Antenna’s Diameter	0.1 m
Total AOM Frequency Shift	84 MHz
Pulse Repetition Frequency	20 kHz
Pulse Width	200 ns
Pulse Energy	14 µJ
ADC Sampling Rate	400 MHz
ADC Number of Bits	14-bits
Detector’s Type	Balanced Detector

**Table 2:** Number of lags and lag delay (τ) influences on velocity resolution and maximum velocity range.

Number of Lags	Lag Delay τ (ns)	Frequency Res. (MHz)	Velocity Res. (m/s)	Maximum Freq. (MHz)	Maximum Velocity (m/S)
16	10	3.2	2.4	+/− 50	38
8	10	6.6	4.8	+/− 50	38
8	15	4.4	3.4	+/− 32	25
4	10	14.3	10.7	+/− 50	38
FFT	2.5	3.125 (N = 128)	2.34	+/− 200 *	150 *

* Theoretically, the maximum detectable frequency of signals sampled at 400 MHz is a signal with a bandwidth of 200 MHz. Since the AOM frequency shift of the outgoing pulse is shifted by 84 MHz and the detector’s bandwidth is 125 MHz, the maximum detectable Doppler shift is approximately 41 MHz. As a result, the maximum measurable wind velocity is approximately 31 m/s.
